# Anticorrosion behaviour and tribological properties of AZ31 magnesium alloy coated with Nb_2_O_5_/Nb_2_O_5_–Mg/Mg layer by magnetron sputtering†

**DOI:** 10.1039/d2ra04907d

**Published:** 2022-10-07

**Authors:** Ziyu Ding, Qianhong Yuan, Hao Wang, Yinghong Tang, Yimin Tan, Quanguo He

**Affiliations:** School of Packaging and Materials Engineering, Hunan University of Technology Zhuzhou 412007 China csfutanyimin@126.com +86-731-22183858; School of Mechanical Engineering, Hunan University of Technology Zhuzhou 412007 China; School of Life Sciences and Chemistry, Hunan University of Technology Zhuzhou 412007 China hequanguo@126.com

## Abstract

Magnesium alloys are attracting increasing attention for the fabrication of temporary implants because of their superior biodegradability and biocompatibility. However, their high degradation rate under physiological conditions limits their clinical applications. In this work, a Nb_2_O_5_/Nb_2_O_5_–Mg/Mg multilayer coating was prepared on the surface of AZ31 magnesium alloy by magnetron sputtering in order to improve its corrosion resistance. The microstructure and performance of the layers were studied by SEM, AFM, EDS, and XPS, and a scratch tester, nanoindenter, friction tester, and electrochemical workstation, using Nb_2_O_5_ monolayer coating as a control. The results show that these two coatings significantly improved the mechanical, tribological, and anticorrosion performance of AZ31 magnesium alloy. Compared with a Nb_2_O_5_ monolayer coating, the multilayer coating exhibits an increased adhesion by about 10.6 times, and a decreased wear rate and corrosion current density by one order of magnitude, meaning higher damage resistance. This study provides a feasible strategy for enhancing the properties of ceramic layers on magnesium alloys for medical applications.

## Abstract 2

Magnesium alloys are attracting increasing attention for the fabrication of temporary implants because of their superior biodegradability and biocompatibility.

## Graphical abstract

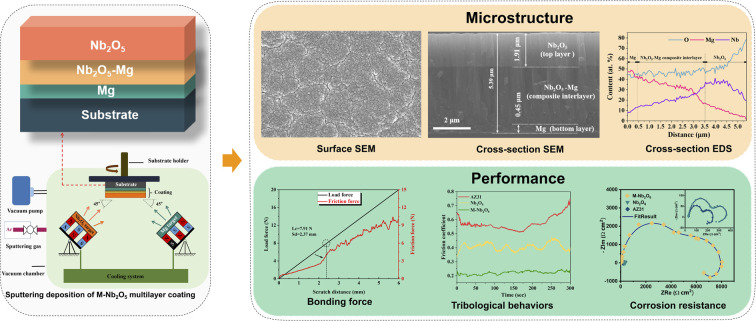

## 1 Introduction

Magnesium alloys are widely studied for their potential as temporary implant materials because of their good biodegradability, biocompatibility, bone conductivity, and for having mechanical performance more similar to natural bone than other implantable materials, such as titanium-based materials and stainless steel.^1–4^ However, the major limitation of magnesium alloys as biomedical materials is their high degradation rate in physiological media.^3^ During service, excessive corrosion of magnesium alloys will cause significant hydrogen evolution and local alkalization, inducing adverse physiological reactions. Moreover, rapid degradation will lead to the loss of mechanical integrity of the implants, prior to tissue healing.^4^

Many methods have been used to control the degradation rate of magnesium alloys. Among these, coating has been demonstrated to be one of the most effective approaches. At present, coating techniques used to modify Mg alloy for corrosion control mainly include physical vapor deposition (PVD),^5^ sol–gel,^6^ micro-arc oxidation,^7^ and plasma electrolytic oxidation.^8^ Among these, sputtering deposition, a commonly used PVD method of coating fabrication, can produce a coating consisting of different materials, including inorganic, organic, and metals. Coatings with different structures can also be produced, such as a monolayer, bilayer, and multilayer. Moreover, the films prepared by sputtering techniques are advantageous, having high compactness, excellent uniformity, strong adhesive force, and controllable structure/composition under a low-temperature preparation environment.^9^

Niobium pentoxide (Nb_2_O_5_) ceramic is a promising coating material for modifying medical implants due to its excellent chemical inertness and biocompatibility. It is reported that Nb_2_O_5_ coating can significantly increase the corrosion resistance of metal implant materials, including magnesium alloys, titanium alloys, and stainless steel.^10–12^ Nb_2_O_5_ coating can promote hydroxyapatite formation, cell adhesion, differentiation, and proliferation, and improve alkaline phosphatase activity.^13,14^ Compared with metal substrates such as 316L^12^ and Ti6Al4V,^15^ Nb_2_O_5_ coating has lower cytotoxicity and lesser induced inflammation. In addition, Nb_2_O_5_ is an effective anti-allergy layer in the prosthesis.^16^ Also, the Nb_2_O_5_ layer shows excellent corrosion resistance and biocompatibility, compared to metal oxide films, such as TiO_2_ and ZrO_2_.^17^ Therefore, various research results recommend that Nb_2_O_5_ be used as a material for surface modification in medical implant fields. However, because of the mismatch between the properties of hard Nb_2_O_5_ ceramics and soft magnesium alloys, especially the coefficient of thermal expansion, if Nb_2_O_5_ is directly deposited on magnesium alloy, there will be considerable coating-substrate interface stress and coating internal stress, resulting in the coating having poor bonding performance.^18^ Under external load, poor adhesion will cause the coating to crack, experience delamination, or even entirely fall off. For clinical applications, the failure of coat-matrix adhesion is unacceptable, as it results in the loss of the properties of the coating, including anti-corrosion, anti-wear, and biocompatibility, conferred to the implant by the layer. More seriously, it releases debris into the surrounding tissue, harming the patient.^19^

It is widely known that introducing an intermediate layer between the coating and the substrate is an effective method for improving the bonding performance of the coating. It has been proven that the intermediate layers can achieve the gradient variation of the component concentration from the substrate to the coating, decreasing the properties mismatch between the film and the matrix, allowing reduced interface stress and reduced internal stress of the layer.^20^ In addition, they can combine the mechanical performance of the film/matrix system and enhance the ability of the layer to resist plastic deformation and wear.^21^ They can serve as a barrier to prevent the occurrence of thickness-throughout defects in the layer, and hinder the passage of corrosive media attacking the matrix, offering outstanding protection for the matrix.^22^ Although introducing an intermediate layer can give the coating many expected properties, the research concerning the coatings containing interlayers on the surface of magnesium alloy is minimal. In addition, there is no report on the microstructure and properties of Nb_2_O_5_ multilayer coating. Therefore, this work produced a Nb_2_O_5_/Nb_2_O_5_–Mg/Mg multilayer coating (code M-Nb_2_O_5_) by sputtering deposition, and investigated the microstructure, adhesion, mechanical, anti-wear, and anti-corrosion properties of the coating, with a Nb_2_O_5_ monolayer coating as a control. It aims to evaluate the effect of introduced interlayers, on the structure and performance of Nb_2_O_5_ coating, to provide a reference for improving the properties of ceramic coating AZ31.

## 2 Materials and methods

### 2.1 Material

AZ31 magnesium alloy plates (Al 2.8–3.5 wt%, Zn 0.7–1.11 wt%, Mn 0.2–0.4 wt%, Fe 0.1–0.2 wt%, Si and Cu < 0.05 wt%, and the surplus, Mg), produced by Dongguan JuBao Co., Ltd, China, and Si wafers (10 × 10 × 0.5 mm, 〈100〉 orientation), manufactured by Dongguan Senshuo Co., Ltd, China, were used as the substrate materials. The AZ31 matrix sheets (with dimensions of 10 × 10 × 2 mm) were mechanically polished with 4000 mesh silicon carbide sandpaper and 500 nm alumina solution to gain a mirror-like effect (Shanghai Grinding Wheel Co., Ltd, China). Afterwards, they were washed in ethanol for 2 min using an ultrasonic cleaner (KQ-50DB, Kunshan Ultrasonic Instrument Co., Ltd, China), then vacuum dried for 20 min. Nb_2_O_5_ ceramic and Mg metal targets (3 inches diameter, 99.99% purity), purchased from Beijing ZNXC Co., Ltd, China, acted as the deposition sources. Argon (99.99% purity) was used as the sputtering gas.

### 2.2 Coating deposition

The M-Nb_2_O_5_ multilayer coating comprises three layers, shown in Fig. S1,† where the bottom layer is the pure Mg metal film used as the bond layer. The composite intermediate layer of Nb_2_O_5_–Mg is used as the transition layer, to decrease the CTE mismatch between the Nb_2_O_5_ layer and AZ31 substrate, so as to enhance adhesion. The top layer is the pure Nb_2_O_5_ ceramic film, which acts as the function layer, to improve the properties of the AZ31 substrate, such as its mechanical, anti-corrosion, and anti-wear performance. In most engineering applications, a protective layer with a thickness of 2–5 μm will be sufficient^23^ and has excellent adhesive and anti-corrosion properties, with a bond layer of 0.3–0.6 μm thickness.^24,25^ So, in this work, a total thickness of 5.0 μm is used for the M-Nb_2_O_5_ multilayer coating, and 2 and 0.4 μm were used as the thickness of the outer layer of Nb_2_O_5_ and bond layer of Mg respectively.

A magnetron sputtering equipment (JP450, China), equipped with two targets in confocal installation mode, was used to prepare the M-Nb_2_O_5_ multilayer coating, displayed in Fig. S2.† Before the film production, the matrix and targets were each sputter-cleaned with Ar^+^ ions for 20 min, to remove surface impurities under a radio frequency (RF) power of 200 W, an Ar gas flow of 20 sccm and a background pressure of 1 × 10^−3^ Pa. The M-Nb_2_O_5_ multilayer coating was synthesized on the substrates by successively producing an Mg metal layer, Nb_2_O_5_–Mg ceramic-metal composite layer, and Nb_2_O_5_ ceramic layer. Mg and Nb_2_O_5_ layers were deposited using the direct current (DC) sputtering mode at 60 W and the RF sputtering mode at 250 W respectively. The Nb_2_O_5_–Mg composite layer was synthesized *via* RF and DC co-sputtering mode. During layer deposition, the matrix was 75 mm from the target and rotated at 20 rpm. It was not heated. The coating on the Si wafer was used to investigate the layer's cross-section microstructure. The coated AZ31 alloys were used to assess their performance, including adhesion, mechanical properties, tribological behavior, and corrosion resistance. A monolayer Nb_2_O_5_ film was deposited on the AZ31 matrix as a control. The detailed parameters for synthesizing M-Nb_2_O_5_ multilayer coating are given in Table S1.†

### 2.3 Coatings characterization

The surface, cross-sectional morphology, and chemical composition were detected using SEM (SU8000, Japan) configured with EDS. The surface three-dimensional appearance and roughness was evaluated by AFM (EasyScan2, Switzerland). An XRD machine (Ultima IV, Japan) with Cu Kα radiation was employed to identify the crystalline structures. The XRD images were measured in the 2*θ* region of 10–80°. The elemental valence states at the surface of the M-Nb_2_O_5_ coating were investigated using XPS (EscaLab 250Xi, US) with Al Kα irradiation. The XPS spectra were calibrated with the C 1s peak at the binding energy of 284.8 eV.

A scratch tester (MFT-4000, China) was used to investigate the bonding performance of the coating. During the test, a diamond indenter (200 μm radius) was gradually scratched the coating surface, by continuously increasing the load. The test was performed in the 0–20 N loading range, with a loading rate of 20 N min^−1^ and a scratch length of 6 mm. The critical normal load (*L*_c_) was applied to evaluate the film adhesion. *L*_c_ is related to the continuous detachment of the coating from the substrate, determined through microscopic observations, combined with scratch curves. The signal curves, including normal load, friction load, and the coefficient of friction (COF), were collected during the experiments. The scratch morphology was observed using SEM.

A nanoindentation (CSM, Switzerland) was used to examine the mechanical properties of the monolayer and multilayer films. The test was performed with a Berkovich indenter, under a maximum load of 2 mN with a loading time of 2 s and a residence time of 2 s for the full load. The hardness (*H*) and elastic modulus (*E*) were calculated *via* the Oliver–Pharr method, using the load–displacement curves, and averaged. Five tests were carried out, to get the mean value for each sample.

The tribological behavior was investigated by a reciprocating wear tester (UMT-2, US) at room temperature. During the tests, a constant normal load of 1 N was applied to the GCr15 steel ball (*ϕ* 9.525 mm), with no lubrication. The sliding speed of the specimen relative to the steel ball was 6 mm s^−1^, with a track length of 6 mm for 300 s. The surface morphology and wear profile of the wear tracks were observed by SEM and 3D profiler (KH-7700, Japan) respectively. The wear ratio (*w*) was calculated by eqn (1):^26^1
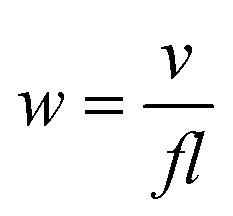
where *v*, *f*, and *l* stand for the volume attrition (mm^3^), normal load (N), and sliding distance (m) respectively.

The electrochemical behavior of naked AZ31 alloy, Nb_2_O_5_, and M-Nb_2_O_5_ coating samples was investigated by potentiodynamic polarization (PDP) and electrochemical impedance spectroscopy (EIS) experiments in simulated body fluids (SBF) at 37 ± 0.5 °C. All measurements were performed on an electrochemical detection equipment (SP-15/20A, France), using the traditional three-electrode cell system. The sample was used as the working electrode, with an area of 1 cm^2^ exposed to the electrolyte. Saturated Ag/AgCl and a platinum sheet were used as the reference and counter electrodes. The SBF solution had a PH of 7.4 and was prepared according to the suggestions of Tadashi Kokubo *et al.*^27^ Before the electrochemical measurements, the open circuit potential (OCP) was observed for 30 min, to allow the system to achieve an equilibrium in the SBF solution. EIS was conducted in the frequency range from 10^5^ Hz to 10^−2^ Hz, at OCP with an AC amplitude of 10 mV. PDP tests were done after EIS tests at a scan rate of 1 mV s^−1^ from 250 mV below OCP to 500 mV above. Each sample was measured five times, and the outcomes were averaged. The surface morphology of the coatings after the polarization test was analyzed by SEM.

## 3 Results and discussion

### 3.1 Micro-characteristics of the coatings

**Fig. 1:**
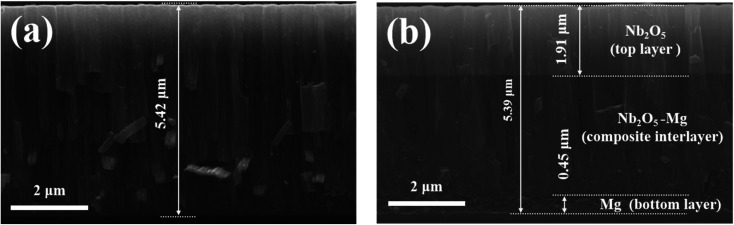
SEM sectional micrographs of coated Si specimens: (a) Nb_2_O_5_, (b) M-Nb_2_O_5_.

Fig. 1 gives the fractured cross-section SEM patterns of the Nb_2_O_5_ and M-Nb_2_O_5_ coated samples. Two coatings show a columnar structure growth model, a common characteristic of coatings produced at low temperature, *via* the magnetron sputtering method.^21^ The total thickness of the Nb_2_O_5_ and M-Nb_2_O_5_ coating is about 5.42 μm and 5.39 μm respectively. Both coating samples have a distinct interface between the layer and substrate. Still, the bonding interface between the Mg bond layer and Nb_2_O_5_–Mg composite interlayer is unclear, indicating a gradual variation in element concentration rather than an abrupt change. Based on the structural composition of the M-Nb_2_O_5_ multilayer coating and the sputtering rate of each layer, it can be determined that the Mg bond layer has a thickness of ∼0.45 μm, and the thicknesses of the Nb_2_O_5_–Mg composite interlayers and the Nb_2_O_5_ top layer are about 3.03 μm and 1.91 μm respectively. In addition, the cross-sectional morphology of the two coating samples is different. The Nb_2_O_5_ monolayer coating appears to have a noticeably bigger column width than M-Nb_2_O_5_ because of its large thickness. Although the thickness of the M-Nb_2_O_5_ multilayer coating is very close to that of the Nb_2_O_5_ monolayer, it has a smaller column width and a denser structure than the Nb_2_O_5_ layer, which could be because the interfaces of multiple interlayers disrupt the column continuity and the Mg-doped material in the interlayers fills the pinholes and voids.^28^

The quantitative evaluation of cross-sectional components in the layers is done by EDS line scanning, as shown in Fig. S3.† As shown, the Nb_2_O_5_ monolayer and M-Nb_2_O_5_ multilayer coating exhibit different characteristics in the curve of variation in element content with thickness. The concentrations of Nb and O elements in Nb_2_O_5_ monolayer coating are relatively stable over the whole thickness. In contrast, along the growth direction of the layer, the M-Nb_2_O_5_ composite coating shows a gradual increase in the contents of Nb and O elements, together with a continuous reduction of Mg. It is worth noting that the Nb content decreases when moving closer to the surface. This is because the coating adsorbs oxygen and water molecules in the air, which can be confirmed by subsequent XPS detection. Besides, the M-Nb_2_O_5_ multilayer film exhibits lower Nb and O contents at the coating-substrate bonding interface. According to previous study,^29^ a low composition gradient at the coating-substrate bonding interface and the absence of abrupt changes in the composition contents between adjacent layers are conducive to reducing interface stress and enhancing the adhesion of the coating. The results predict that the M-Nb_2_O_5_ multilayer film would show a higher adhesion to the AZ31 matrix than the pure Nb_2_O_5_ film.

**Fig. 2:**
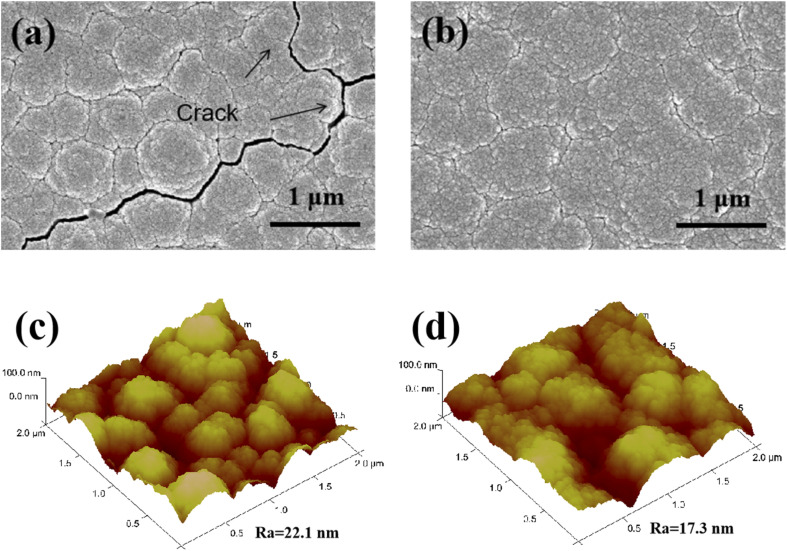
SEM and 3D AFM images of the coating samples: (a) Nb_2_O_5_, (b) M-Nb_2_O_5_.

Fig. 2 shows the SEM and 3D AFM images of Nb_2_O_5_ and M-Nb_2_O_5_ coated sample surfaces. The SEM images (Fig. 2a and b) depict that both coatings have a cauliflower-like appearance. However, the structure of M-Nb_2_O_5_ multilayer coating is dense and uniform, while the Nb_2_O_5_ monolayer coating has obvious cracks, indicating an inadequate cohesive strength. Previous studies^30^ demonstrated that a thicker ceramic singer-layer coating on metal substrates has considerably more internal stress and coating-matrix interface stress, resulting in low cohesive force and adhesive force for the layers. The Nb_2_O_5_ monolayer film sample has a thicker Nb_2_O_5_ layer than the M-Nb_2_O_5_ multilayer coating samples (Fig. 1). Thus, it is easily cracked.

From AFM images (Fig. 2c and d), the surface roughness (*R*_a_) measured from Nb_2_O_5_ and M-Nb_2_O_5_ samples are 22.1 nm and 17.3 nm, which is connected to the thickness of the Nb_2_O_5_ layer. As the growth model of the sputtered film has inverted conical columnar features, a thicker film has a wider columnar size, with larger hats on the surface, thus allowing larger *R*_a_ values.^31^ Although the M-Nb_2_O_5_ multilayer coating has almost the same thickness as the Nb_2_O_5_ monolayer coating, the Nb_2_O_5_–Mg/Mg composite layer blocks the continuous growth of the columnar structure. In addition, the thickness of the outer layer of Nb_2_O_5_ film is smaller than the Nb_2_O_5_ monolayer coating, thus showing a lower *R*_a_ value relative to the Nb_2_O_5_ monolayer coating.

The EDS spectra (Fig. S4†) illustrate that both layer surfaces contain O, Nb, and Mg elements. The atomic ratio of O to Nb is above 2.5, meaning that other oxygen compounds could appear, which can be confirmed by subsequent XPS analysis (Fig. S5†). The small amount of detected Mg could come from the AZ31 matrix or interlayer, caused by the coating being too thin or defective and the electron beam penetrating the Nb_2_O_5_ layer. Therefore, the M-Nb_2_O_5_ sample containing Mg in both the substrate and the interlayer has a higher Mg content (about 2.05 at%) than the Nb_2_O_5_ single-layer sample with a thicker Nb_2_O_5_ layer.

**Fig. 3:**
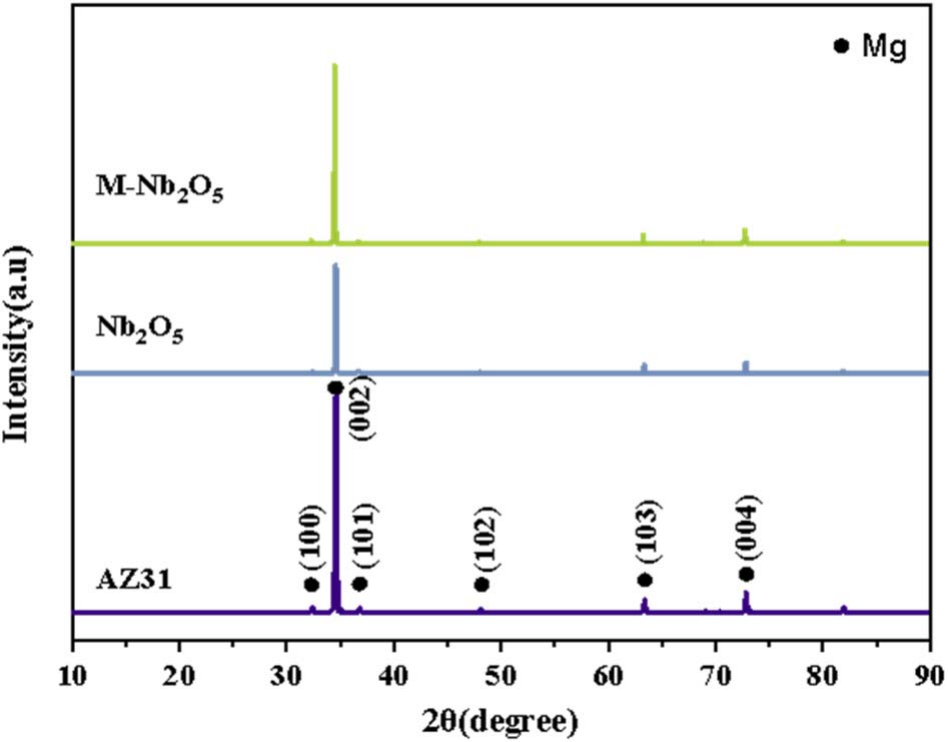
XRD patterns of naked AZ31 and coated AZ31 specimens.

Fig. 3 displays the XRD spectra of unmodified and modified AZ31 alloy specimens. Two coating samples have similar peaks to those of the untreated AZ31 alloy, and no feature peaks associated with Nb_2_O_5_, implying an amorphous structure. It has been found that Nb_2_O_5_ has multiple crystalline phases, including hexagonal and orthorhombic phases, mainly affected by deposition method and annealing temperature. For example, Rosenfeld *et al.*^32^ discovered that the Nb_2_O_5_ coating deposited by magnetron sputtering crystallized at 500 °C with a few weak hexagonal phase peaks. The intensities of the peak increased continuously with the temperature. When the temperature reached 700 °C, an orthogonal phase was discovered. In another piece of research, Dinu *et al.*^33^ found that the Nb_2_O_5_ film on Ti6Al4V alloy fabricated with electron beam technique, exhibited an amorphous structure. The coating heated at 600 °C showed hexagonal and orthogonal phases, and at 800 °C, the peak intensities strengthened, and the crystal size increased from 70 nm to 78 nm.

The XPS spectra for M-Nb_2_O_5_ coated AZ31 alloy was shown in Fig. S5.† The XPS survey spectrum (Fig. S5a†) illustrated that the M-Nb_2_O_5_ sample surface contains Nb, Mg, O, and C elements. The XPS core spectra of Nb 3d show two peaks (Fig. S5b†). The peaks at 207.1 eV and 209.9 eV are attributed to the Nb 3d_5/2_ and Nb 3d_3/2_, indicating the existence of Nb_2_O_5_.^34^ The band shift of Nb 3d_5/2_ and Nb 3d_3/2_ is 2.8 eV, showing no difference between these findings and existing literature.^35^ From Fig. S5c,† it is found that the Mg 1s can be divided into two peaks. The A1 peak (1302.9 eV) belongs to Mg(OH)_2_, whereas the A2 peak (1303.9 eV) comes from MgO.^36^ The binding energy peaks of the O 1s core spectrum in Fig. S5d† have three de-convoluted peaks. The peaks at 532.6 eV (L1) and 531.4 eV (L2) correspond to O 1s in MgO and Mg(OH)_2_ respectively, whereas O 1s at the L3 peak (530.1 eV) is related to Nb_2_O_5_. The above XPS results indicate the presence of Nb_2_O_5_, MgO, and Mg(OH)_2_ on the surface of M-Nb_2_O_5_ coated AZ31 alloy.

### 3.2 Adhesion strength

**Fig. 4:**
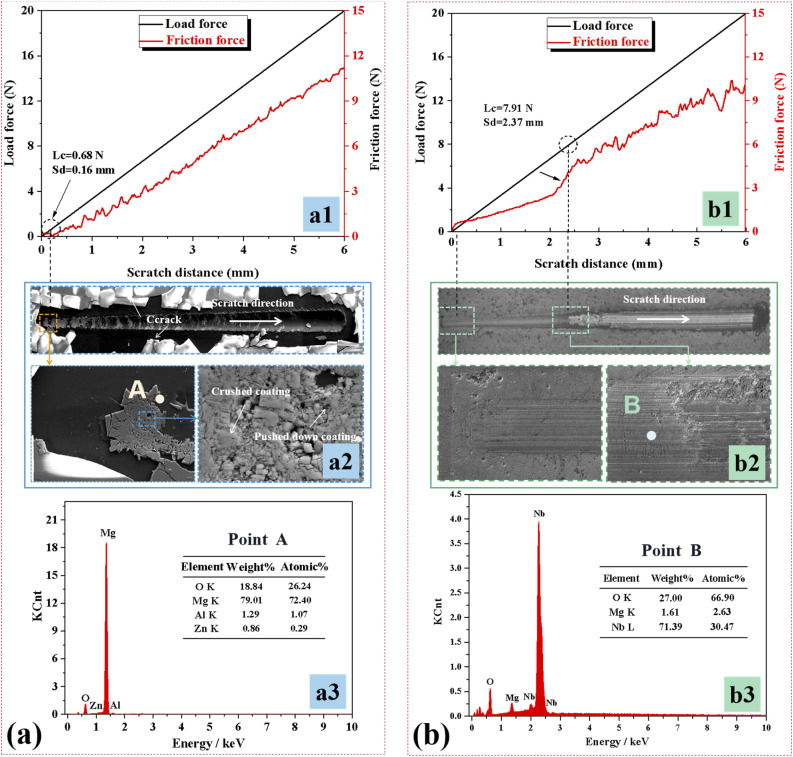
Scratch curves, morphologies of scratch track, and the EDS spectrum taken from the specified location for the studied coatings: (a) Nb_2_O_5_, (b) M-Nb_2_O_5_.

The scratch test results of Nb_2_O_5_ and M-Nb_2_O_5_ treated AZ31 specimens are displayed in Fig. 4. The results show that the external load increases linearly with the sliding distance, and the friction oscillation enlarges. Fig. 4a2 shows that the film around the scratch path of the Nb_2_O_5_ monolayer coating fell off or cracked, indicating poor adhesion. Since the mismatch in thermal expansion coefficient between ceramic layer and metal substrate would allow the thick layer to have a high interface and internal stress,^23^ the thick Nb_2_O_5_ monolayer coating is prone to delamination and cracking caused by pressing as the applied load increases. As shown, the film at the edges throughout the scratch was detached entirely, so it is difficult to assess its adhesive force, based on the characteristics of the scratch track. Still, by observing Fig. 4a1, it is determined that the matrix exposed at position A in the scratch track, with a scratch length of 0.16 mm, is connected to the matrix exposed along both sides of the scratch path. It could be suggested that the film fails from here. From the scratch curve (shown in Fig. 4a1), the load force at this time is about 0.68 N, thought to be the adhesion of Nb_2_O_5_ monolayer coating.

Fig. 4b shows that the EDS results of the M-Nb_2_O_5_ multilayer film at position B contains Nb, O, and Mg elements, indicating that the film has not failed. Subsequently, the film undergoes continuous peeling off at a scratch length of 2.37 mm, under an applied force of 7.91 N (shown in Fig. 4b1 and b2), implying an adhesion of 7.91 N. This is more than 11 times stronger than the Nb_2_O_5_ monolayer coating. The scratch results demonstrate that the multilayer structure can significantly increase the adhesive strength between the Nb_2_O_5_ layer and AZ31 alloy substrate. The augmentation in adhesive property for the M-Nb_2_O_5_ multilayer is associated with a stronger interface effect, and greater affinity between the Nb_2_O_5_ film and the Nb_2_O_5_–Mg film, and between the Mg film and the AZ31 magnesium alloy matrix than those between the Nb_2_O_5_ film and the AZ31 magnesium alloy matrix. This could contribute to achieving metallurgical bonding with solid bonding properties.^29^ Besides, incorporating Nb_2_O_5_–Mg/Mg double interlayers allows a gradient variation in the composition between the Nb_2_O_5_ coating and AZ31 substrate, which would be conducive to reducing the performance difference between them, especially the CTE mismatch. This causes a reduced residual thermal stress. To quantitatively assess the structure's effect on the layer's residual stress, the residual stress distribution in coatings was investigated by finite element modeling (FEM), using commercial software ANSYS. The FEM model and boundary conditions used by Ding *et al.*,^29^ were adopted in the analysis. Table S2† lists the material properties adopted for the FES. Fig. S6† shows the results of the FEM. The M-Nb_2_O_5_ film has a maximum residual stress of 62.8 MPa, 22.9% smaller relative to the Nb_2_O_5_ single-layer coating (81.5 MPa). Decreased residual stress can reduce the risk of coating cracking and delamination under applied load. Hence, the M-Nb_2_O_5_ multilayer coating has superior adhesive performance compared to the Nb_2_O_5_ monolayer coating.

### 3.3 Mechanical properties

Fig. S7† depicts the load-indentation depth curves of naked, Nb_2_O_5_, and M-Nb_2_O_5_ coated AZ31 specimens, obtained by nano-indentation. The load-depth curve is continuous, meaning there was no sudden cracking and delamination of the coating during the test. The Nb_2_O_5_ and M-Nb_2_O_5_ coated specimens exhibit a maximum indentation depth of approximately 198 nm and 159 nm, respectively, which are smaller than that of the bare AZ31 alloy (∼285 nm), suggesting an increased local plastic deformation resistance. Moreover, the film samples show a maximum indentation depth of less than one-tenth of the layer depth, indicating that the substrate's effect on the experimental outcomes could be ignored. The hardness (*H*) and elastic modulus (*E*) obtained from the curves are listed in Table 1. Both coating specimens exhibit significantly bigger *H* and *E* values than the bare AZ31 alloy, indicating that the layer can boost the mechanical performance of AZ31. The *H* (3.2 GPa) and *E* (83.2 GPa) of the M-Nb_2_O_5_ multilayer sample were 52.4% and 47.5% higher than the Nb_2_O_5_ monolayer sample. The enhancement of mechanical properties of M-Nb_2_O_5_ film is associated with the formation of the multilayered interfaces and gradient components after introducing Nb_2_O_5_–Mg/Mg composite interlayer. The multilayered interfaces could reduce the amounts and the movability of dislocations in the Nb_2_O_5_ coating. The gradient composition could decrease the interface stress between the Nb_2_O_5_ layer and the substrate and enhance the interface bonding. Similar results were found in previous studies.^21^ In addition, the *H*/*E* and *H*^3^/*E*^2^ values used to evaluate the resistance to wear and plastic deformation of the layers respectively, are also summarized in Table 1. In general, the larger *H*/*E* and *H*^3^/*E*^2^ coatings show a stronger ability to resist plastic deformation and an excellent anti-wear performance, compared to layers with smaller *H*/*E* and *H*^3^/*E*^2^.^37,38^ From Table 1, the *H*/*E* (38.5 GPa) and *H*^3^/*E*^2^ (4.7 GPa) of the M-Nb_2_O_5_ coated AZ31 sample were 3.5% and 62% higher than the Nb_2_O_5_ coated AZ31 sample, meaning stronger crack resistance and wear resistance.

**Table 1:** Mechanical and tribological performance of naked and coated AZ31 specimens

Specimen	AZ31	Nb_2_O_5_	M-Nb_2_O_5_
*H* (GPa)	0.9	2.1	3.2
*E* (GPa)	42.9	56.4	83.2
*H*/*E*	21	37.2	38.5
*H* ^3^/*E*^2^ (GPa)	0.4	2.9	4.7
Mean COF	0.574	0.408	0.221
Wear rate (mm^3^ N m^−1^)	2.524 × 10^−3^	1.351 × 10^−3^	0.044 × 10^−3^

### 3.4 Tribological behavior

Fig. S8† gives the coefficient of friction (COF) curves of untreated AZ31, Nb_2_O_5_, and M-Nb_2_O_5_ coated AZ31 samples with a 300 s moving time. As shown, the COF of AZ31 alloy exhibits a variation in characteristics, from a rapid decline to a gradual increase. The initial rapid drop could be attributed to the quick wear of the oxide films on the AZ31 alloy surface. Then, the COF gradually rises because of the increased friction area, debris growth, and the substrate being softened by friction heat. For Nb_2_O_5_ coated AZ31, the COF exhibits a rapid rise at the initial friction stage, resulting from surface features, such as bulges and pits. After running in, the COF oscillates within an extensive range (0.37–0.47), indicating severe surface damage. It should be noted that, during the testing time of 300 s, the COF of the M-Nb_2_O_5_ coating specimen had the narrowest variation range (0.19–0.25) and the lowest mean value, about 0.221, compared to the naked and Nb_2_O_5_ coated AZ31 specimens. Films with a large *R*_a_ value have a high COF.^39^ At the beginning of the friction test, the Nb_2_O_5_ coating specimen has higher COF than the M-Nb_2_O_5_ coating specimen, because of its bigger *R*_a_ value presented in Fig. 2. However, the coating's risk of delamination, cracking, and shedding may grow with sliding time. Films with large *H*/*E* and *H*^3^/*E*^2^ usually have a good ability to resist plastic deformation and wear, allowing a relatively stable and small COF.^40^ So, the M-Nb_2_O_5_ multilayer coating sample shows a lower average COF than the Nb_2_O_5_ monolayer coating sample, which can be attributed to its high *H*/*E* and *H*^3^/*E*^2^ values.

The wear rates of bare AZ31 and coated AZ31 specimens are presented in Table 1. AZ31 alloy has a wear rate of 2.524 × 10^−3^ mm^3^ N m^−1^, much higher than all coated AZ31 alloys. By comparison, the M-Nb_2_O_5_ coated AZ31 sample has the lowest wear rate of 0.044 × 10^−3^ mm^3^ N m^−1^, 96.7% smaller than the Nb_2_O_5_ coated AZ31 alloy specimens, implying a better wear resistance, which agrees with the results predicted by indentation measurements.

**Fig. 5:**
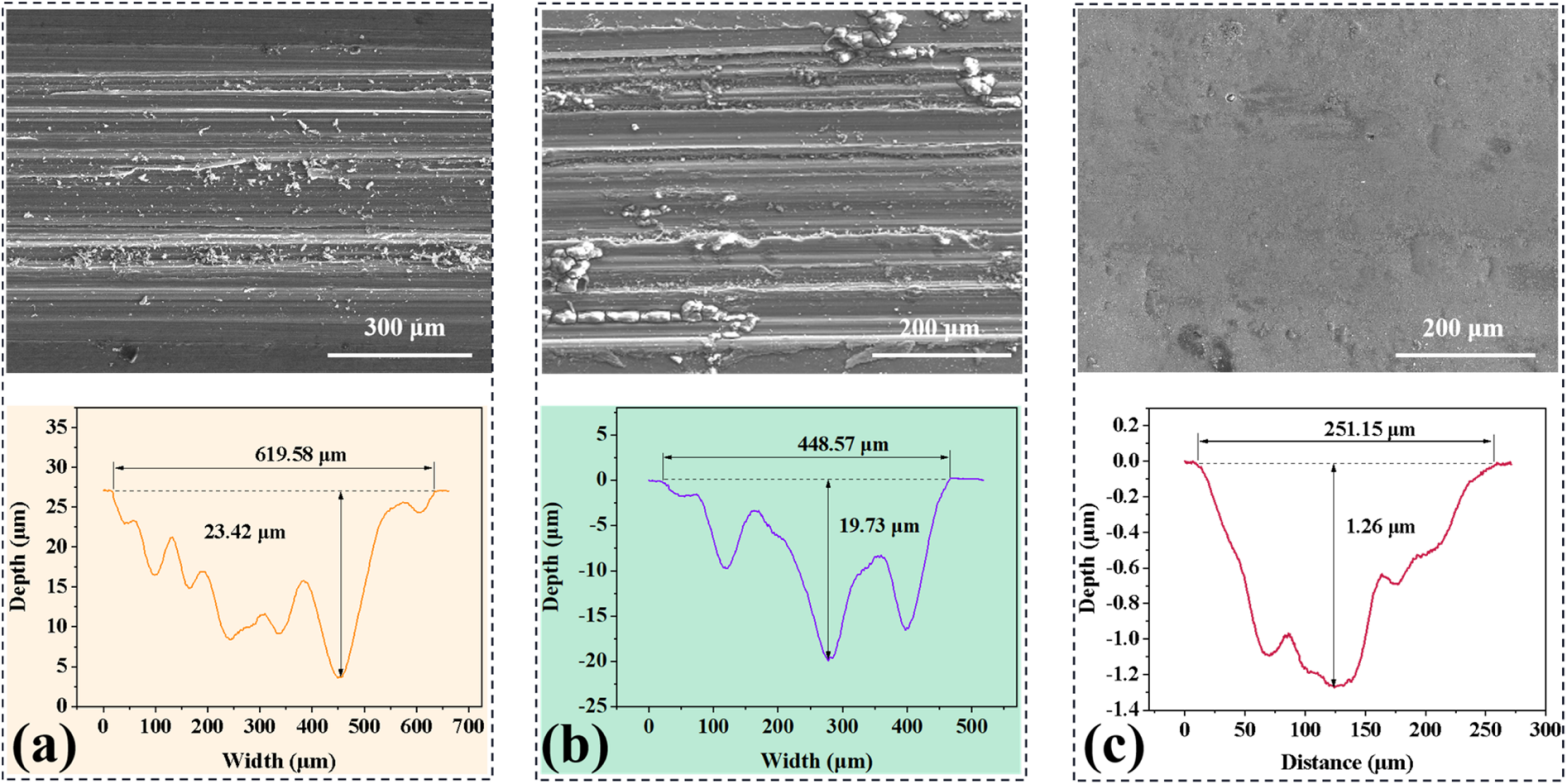
SEM pictures and section profiles of the wear track of specimens: (a) naked AZ31, (b) Nb_2_O_5_ and (c) M-Nb_2_O_5_.

Fig. 5 presents typical SEM photos and section contours of the worn surface of the bare AZ31, Nb_2_O_5_ and M-Nb_2_O_5_ modified AZ31 samples, after the 300 s sliding experiments with no lubricant. As presented in Fig. 5a and b, the worn surface of untreated and Nb_2_O_5_ treated AZ31 samples show many grooves and scratches parallel to the movement direction, meaning that abrasive wear and micro-cutting are their primary wear behaviors. The maximum measured depth of wear track for the Nb_2_O_5_ monolayer coating specimen is approximately 19.73 μm, exceeding its coating thickness, meaning the complete failure of the coating. In contrast, the M-Nb_2_O_5_ coated AZ31 specimen (Fig. 5c) exhibits an almost smooth worn surface, suggesting the primary wear mechanism of grinding and polishing. In addition, it shows a tiny wear depth of 1.26 μm, much less than the coating thickness of 5.39 μm, meaning that the coating was not worn through. The friction experiment outcomes demonstrate that the M-Nb_2_O_5_ multilayer coating has better tribological performance than the Nb_2_O_5_ single-layer coating, and could effectively improve the wear resistance of AZ31 alloy.

### 3.5 Electrochemical behavior

**Fig. 6:**
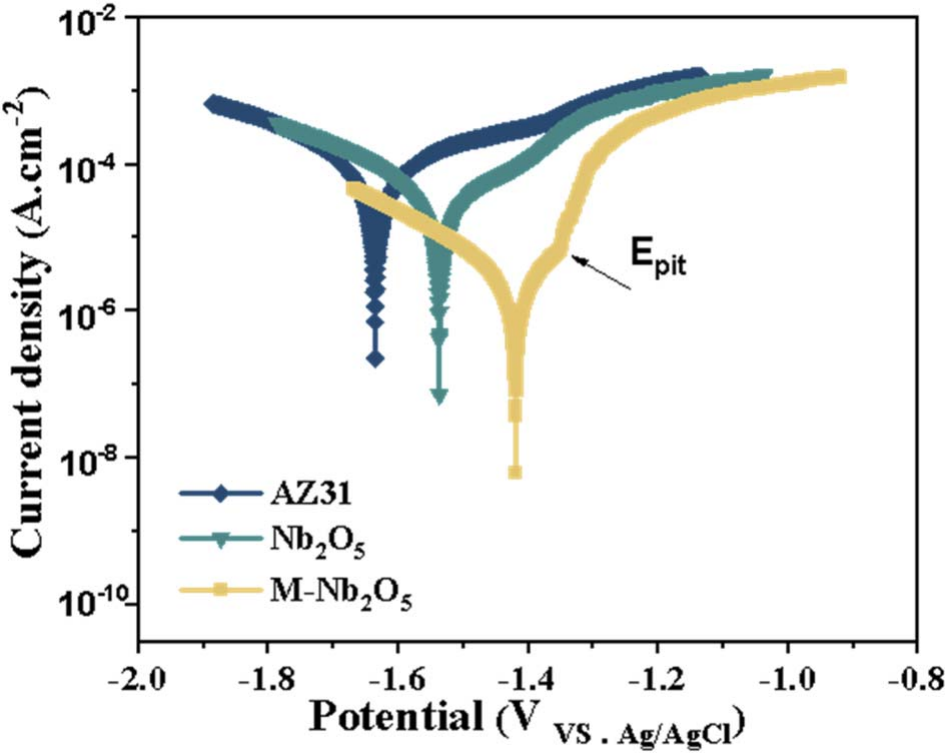
Polarization curves of un-coated and coated AZ31 alloys in SBF solution.

Fig. 6 displays the PDP curves of naked and coated AZ31 specimens in the SBF medium. As shown, the cathodic polarization curve of the M-Nb_2_O_5_ film specimen is below that of uncoated and Nb_2_O_5_-coated AZ31 samples, implying that the multilayer film effectively inhibited cathodic hydrogen evolution.^41^ The current density of the AZ31 in the anode region increases rapidly with the anode potential, indicating that the corrosion was increased. The anodic polarization curves of the Nb_2_O_5_ coating samples were similar to that of the bare AZ31 alloy. They converged with the increase in the anodic potential, implying that the protective effect of the coatings on the AZ31 alloy was limited.^42^ For the M-Nb_2_O_5_ multilayer coating specimen, the anodic polarization curve showed an apparent passive region (from −1.44 V of *E*_corr_ to −1.36 V of *E*_pit_). Moreover, the anodic polarization curve of the M-Nb_2_O_5_ multilayer sample significantly shifts to the region of smaller current density, compared to the Nb_2_O_5_ monolayer sample, indicating reduced magnesium dissolution.

Table S3† presents the corrosion potential (*E*_corr_) and current density (*i*_corr_) of the investigated samples, obtained by fitting the polarization curve using the Tafel extrapolation approach. From the table, all coated AZ31 specimens exhibit increased *E*_corr_ and reduced *i*_corr_ compared with the bare AZ31 alloy, indicating improved corrosion resistance. The M-Nb_2_O_5_ multilayer coating sample has the lowest current density (4.04 × 10^−6^ A cm^−2^), an order of magnitude smaller than bare and Nb_2_O_5_ coated AZ31 alloy samples. In addition, the M-Nb_2_O_5_ multilayer coating sample shows the noblest potential (−1.44 V), which is 190 mV and 100 mV nobler than bare and Nb_2_O_5_ coated AZ31 alloy samples. High *E*_corr_ and low *i*_corr_ of the coating means a small corrosion rate.^43^ The polarization test results demonstrate that the M-Nb_2_O_5_ multilayer coating has a better corrosion protection effect on AZ31 alloy, than the Nb_2_O_5_ monolayer coating.

**Fig. 7:**
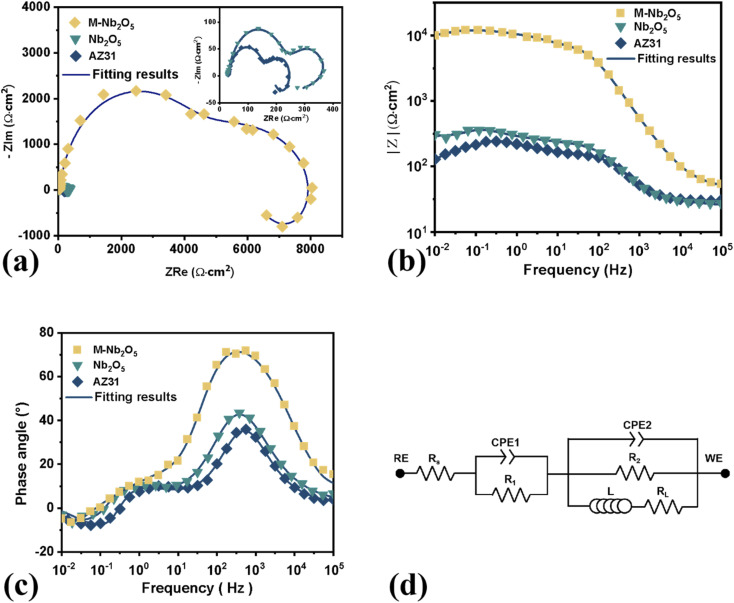
(a) Nyquist plots, (b) Bode impedance and (c) phase angle plots, and (d) corresponding EC of bare AZ31 alloy and coating samples in SBF solution.

EIS experiments were performed to investigate further the corrosion behaviors of the bare and coated AZ31 alloy specimens in the SBF solution. Fig. 7a–c give the Nyquist and Bode diagrams, in which the symbols and solid lines represent experimental and fitting results respectively. As shown, the Nyquist curves for all specimens showed two capacitive loops in the high-frequency and middle-frequency zone, and one inductive loop in the low-frequency zone, in agreement with previous research.^44,45^ It can be found that the M-Nb_2_O_5_ multilayer coating sample had the largest size of capacitive loops, and its impedance (|*Z*|) at the low frequency is more than an order of magnitude bigger than that of the bare AZ31 and Nb_2_O_5_ monolayer coating sample. This outcome implies that the M-Nb_2_O_5_ multilayer film can better boost the anti-corrosion performance of the AZ31 alloy than the monolayer film, consistent with the results of the PDP test.

The equivalent circuit (EC) was employed to fit the impedance spectrum and understand the EIS results, as shown in Fig. 7d. In the EC, *R*_s_ denotes the solution resistance, and CPE_1_ and *R*_1_ are the capacitance and resistance of corrosion products or films on the substrate. CPE_2_ and *R*_2_ represent the constant phase element of double layer and charge-transfer resistance. *L* and *R*_*L*_ are the induction and induction resistance, which could be related to pitting corrosion.^45,46^ Similar equivalent circuits were applied to analyze the corrosion characteristics of untreated and treated AZ31,^44^ ZK60,^47^ and AZ91D^48^ magnesium alloy in the SBF solution. As an evaluation index of anti-corrosion ability, the polarization resistance (*R*_P_) can be calculated by the total sum of *R*_1_ and *R*_2_ (*R*_P_ = *R*_1_ + *R*_2_).^44,45^ The EC shows a good fit in the Nyquist and Bode plot. The relevant results are displayed in Table 2.

**Table 2:** Fitting results of EIS curves for un-coated and coated AZ31 alloy samples in SBF solution

Samples	*R*_s_ (Ω cm^2^)	*Q*_1_ (F cm^−2^ S^*n*−1^)	*R*_1_ (Ω cm^2^)	*n*_1_	*Q*_2_ (F cm^−2^ S^*n*−1^)	*R*_2_ (Ω cm^2^)	*n*_2_	*R*_*L*_ (Ω cm^2^)	*L* (H cm^2^)
AZ31	29.97	1.17 × 10^−3^	7.56 × 10^1^	0.92	1.67 × 10^−5^	1.30 × 10^2^	0.85	3.69 × 10^1^	6.25 × 10^2^
Nb_2_O_5_	24.14	6.30 × 10^−6^	1.51 × 10^2^	0.97	1.74 × 10^−3^	3.21 × 10^2^	0.40	2.34 × 10^2^	4.07 × 10^2^
M-Nb_2_O_5_	29.65	7.06 × 10^−6^	3.60 × 10^3^	0.98	3.63 × 10^−7^	4.82 × 10^3^	0.62	6.60 × 10^3^	4.04 × 10^4^

From Table 2, M-Nb_2_O_5_ multilayer coating samples have a calculated *R*_P_ value of 8.42 × 10^3^ Ω cm^2^, more than about 17 and 40 times higher than that of the M-Nb_2_O_5_ monolayer coating sample and bare AZ31 alloy respectively, indicating that the multilayer coating could be more effective in enhancing the corrosion resistance of AZ31 alloy, than the monolayer film. This improvement can be attributed to its gradual change in composition and multi-layered structure effect. The composition having gradual variation along the deposition direction of the layer, could decrease the internal stress within the coating and the interface stress of the layer/matrix system, thus improving the compactness and adhesion of the coating. In addition, the multi-layered structure could reduce the occurrence of thickness-throughout defects, successfully preventing corrosion media from attacking the substrate, thus significantly enhancing corrosion resistance. By comparison, the pinholes, cracks, and other structural defects in Nb_2_O_5_ single-layer coating provide a channel for corrosive media to pass through the film to the substrate, thereby increasing the exposed area of the substrate, and reducing the protective effect that the coating has on the substrate.

**Fig. 8:**
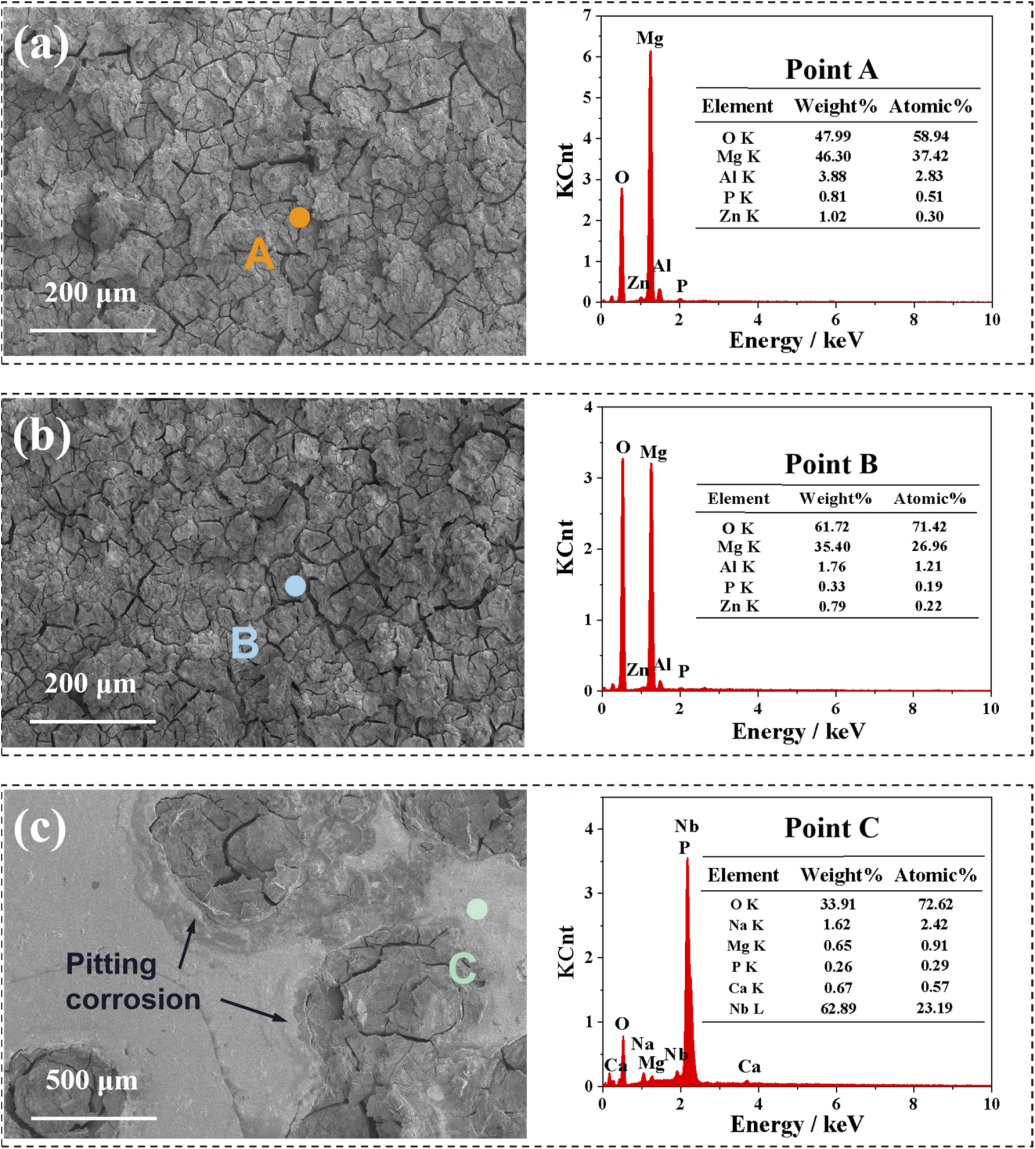
SEM micrographs and EDS results at specific locations of the specimens after polarization experiments: (a) naked AZ31, (b) Nb_2_O_5_ and (c) M-Nb_2_O_5_.

Fig. 8 shows the SEM images and EDS results at specific locations of naked and coated AZ31 specimens, after the electrochemical tests. Before SEM inspection, the corroded samples were ultrasonically cleaned to remove the loose corrosion products adsorbed on the surface of the specimens. It can be seen from Fig. 8a and b that the corroded Nb_2_O_5_ single-layer coating sample show very similar surface characteristics to those of AZ31, that is, rough and cracked, indicating severe damage. By comparison, only a few approximately circular corrosion pits appear on the surface of the M-Nb_2_O_5_ multilayer coating specimen, and most layers are intact. According to the EDS data, position A of AZ31 and position B of Nb_2_O_5_ single-layer coating samples contain the same elements, such as Mg, O, Al, Zn, and P, among which Mg, Al, and Zn come from the substrate, and P from the electrolyte. Nb was not found on the corrosion surface of the Nb_2_O_5_ sample, indicating the complete peeling off of the coating. In addition, the total concentration of Mg and O elements exceeds 95%, indicating that the main corrosion product is Mg(OH)_2_.^49^ For the M-Nb_2_O_5_ multilayer coating sample (shown in Fig. 8c), the position (point C) without corrosion pits contains O, Nb, Na, Mg, P, and Ca, in which the total content of O and Nb elements reaches 95.81 at%, indicating an intact coating in this area. These phenomena show that the corrosion protection effect of M-Nb_2_O_5_ multilayer coating on AZ31 alloy is better than that of Nb_2_O_5_ monolayer coating, consistent with the conclusion of the polarization and impedance tests.

## 4 Conclusions

In this work, Nb_2_O_5_/Nb_2_O_5_–Mg/Mg (code M-Nb_2_O_5_) multilayer coating was deposited on AZ31 magnesium alloy using the magnetron sputtering deposition process. The microstructure, adhesive, mechanical, tribological, and anti-corrosion performance of the coating was studied, with a Nb_2_O_5_ monolayer coating as a control. These two coatings show a cauliflower-like surface morphology and amorphous columnar structure. The M-Nb_2_O_5_ multilayer coating is dense and uniform, while the Nb_2_O_5_ monolayer film cracks significantly. Both layers greatly enhanced the mechanical and tribological properties and corrosion resistance of the AZ31 magnesium alloy. In contrast, multilayer coating exhibited better damage resistance due to its higher adhesion and fewer defects. These experimental results provide a workable strategy for improving the performance of ceramic coating on Mg alloy for medical applications.

## Conflicts of interest

The authors declare no conflict of interest.

## Supplementary Material

RA-012-D2RA04907D-s001
