# Supplementary material for: Anticorrosion behaviour and tribological properties of AZ31 magnesium alloy coated with Nb2O5/Nb2O5–Mg/Mg layer by magnetron sputtering
Source: RSC Adv. 2022 Oct 7;12(43):28196–206. doi: 10.1039/d2ra04907d (PMC9540408; doi:10.1039/d2ra04907d)
Supplement: RA-012-D2RA04907D-s001 [file RA-012-D2RA04907D-s001.pdf]

Electronic Supplementary Material (ESI) for RSC Advances.
This journal is © The Royal Society of Chemistry 2022

# Anticorrosion behaviour and tribological properties of AZ31 magnesium alloy coated with $Nb_2O_5$/$Nb_2O_5$-Mg/Mg layers by magnetron sputtering

Ziyu Ding[a], Qianhong Yuan[b], Hao Wang[b], Yinghong Tang[b], Yimin Tan[a,*], Quanguo He[c,*]

a School of Packaging and Materials Engineering, Hunan University of Technology, Zhuzhou 412007, China
b School of Mechanical Engineering, Hunan University of Technology, Zhuzhou 412007, China
c School of Life Sciences and Chemistry, Hunan University of Technology, Zhuzhou 412007, China

Electronic Supplementary Material (ESI) for RSC Advances.
This journal is © The Royal Society of Chemistry 2022

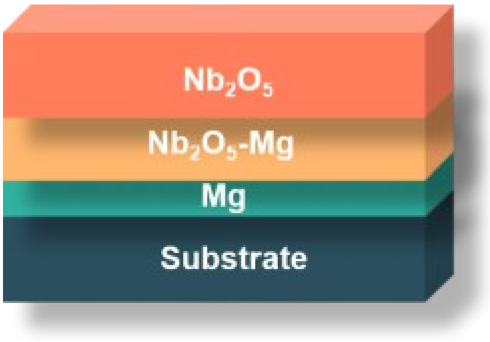


**Figure S1.** Structural model of M-$Nb_2O_5$ multilayer coating.

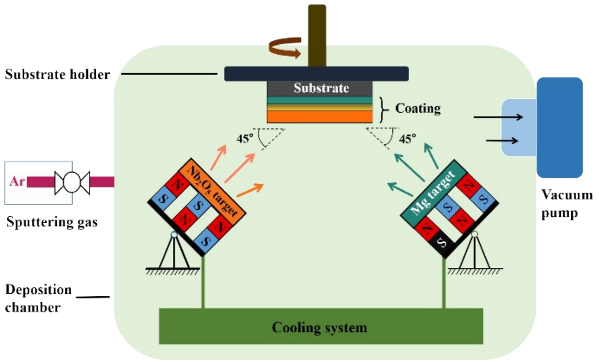


**Figure S2.** Schematic illustration of the magnetron sputtering system.

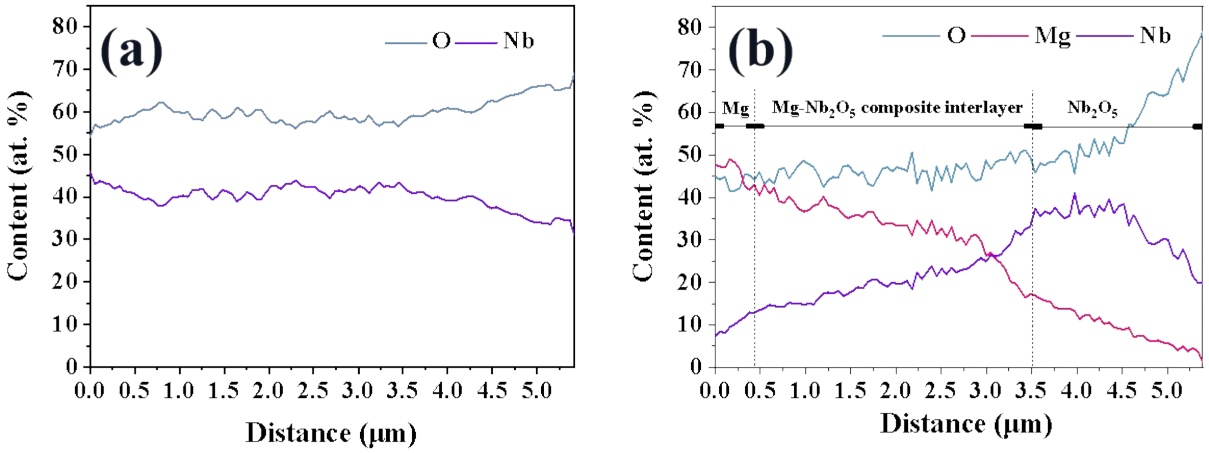


**Figure S3**. Cross-sectional EDS line scanning of the films: (a) $Nb_2O_5$, (b) M-$Nb_2O_5$.

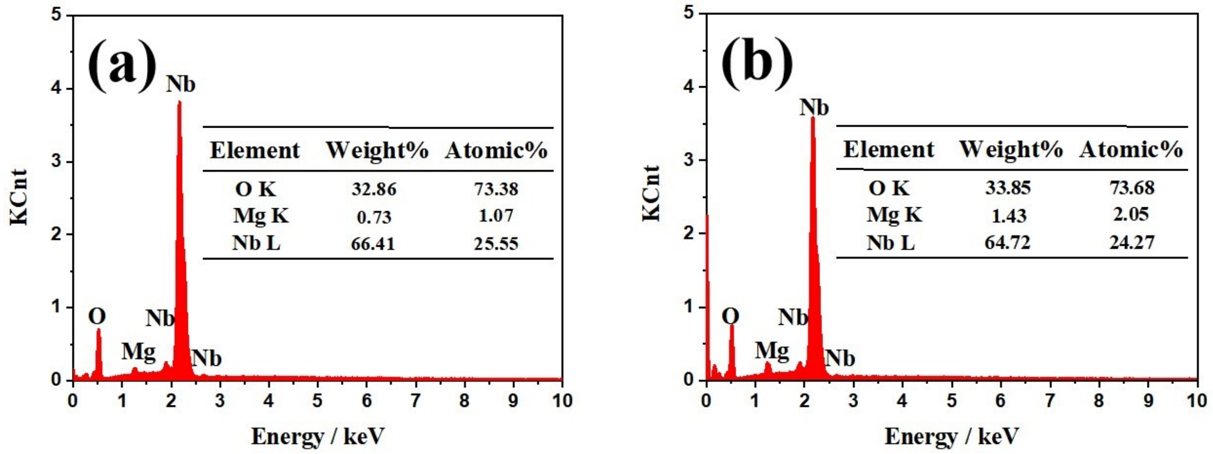


**Figure S4**. Surface EDS analysis of the layers: (a) $Nb_2O_5$ , (b) M-$Nb_2O_5$.

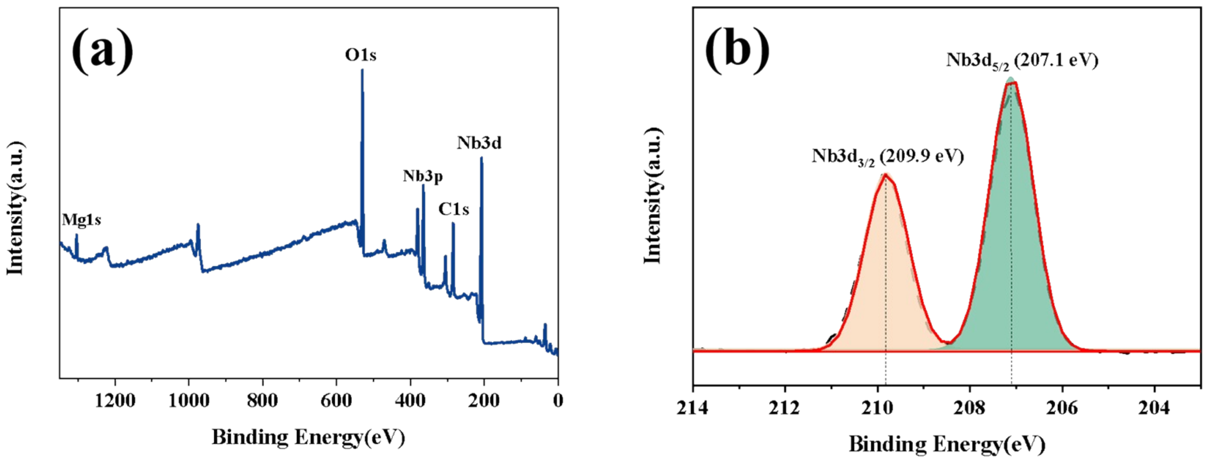

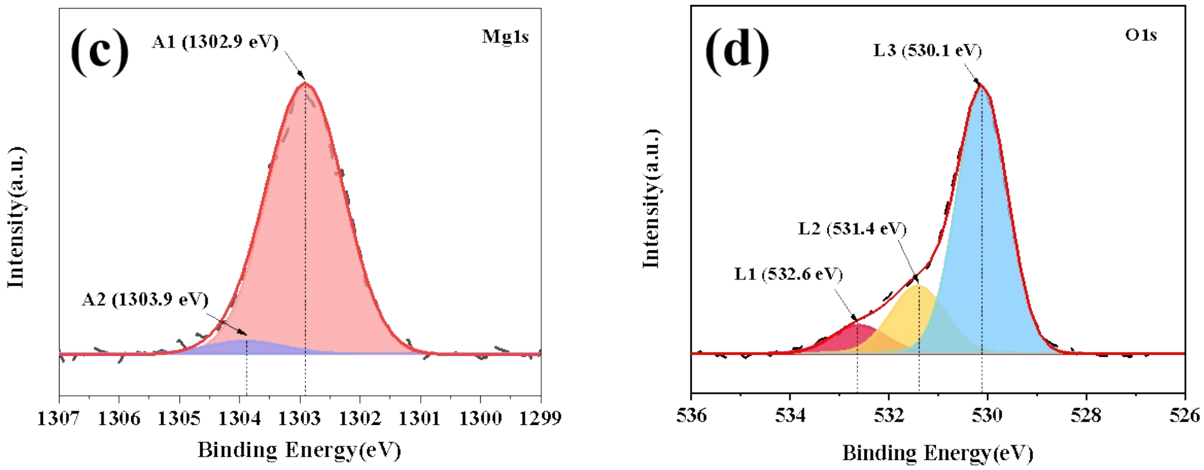


**Figure S5**. (a) XPS survey spectrum of M-$Nb_2O_5$ coating and fine spectra of (b) Nb 3d, (c) Mg 1s and (d) O 1s.

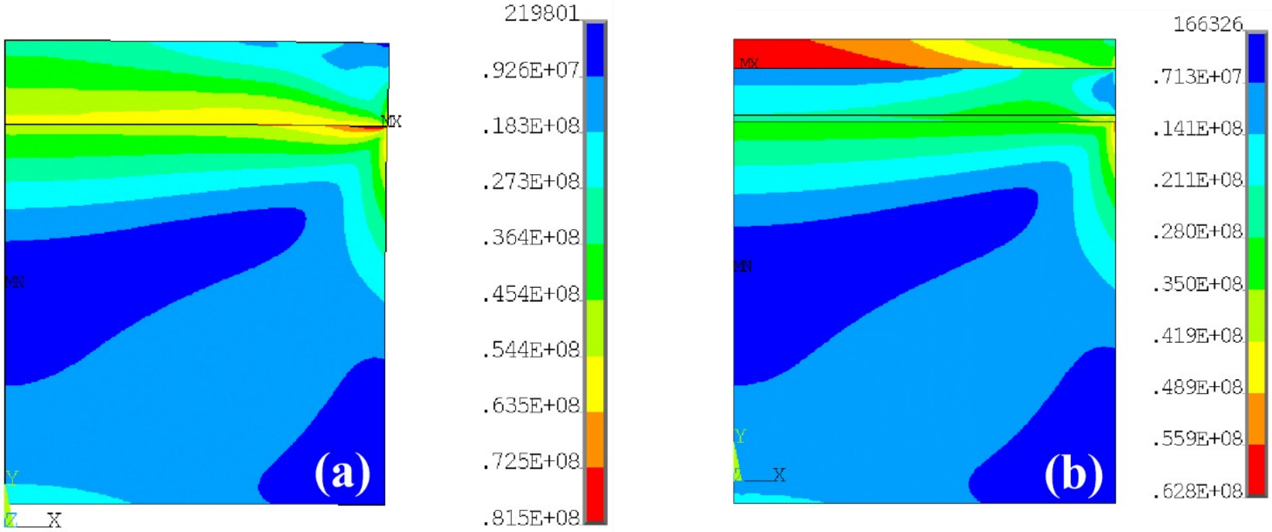


**Figure S6**. FEM results of residual stress distribution in the coatings: (a) $Nb_2O_5$ and (b)M-$Nb_2O_5$.

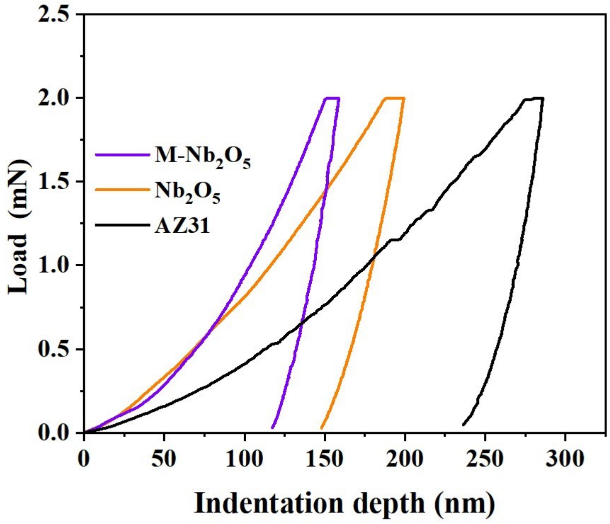


**Figure S7**. Load-indentation depth curves of naked, $Nb_2O_5$ and M-$Nb_2O_5$ coated AZ31 specimens.

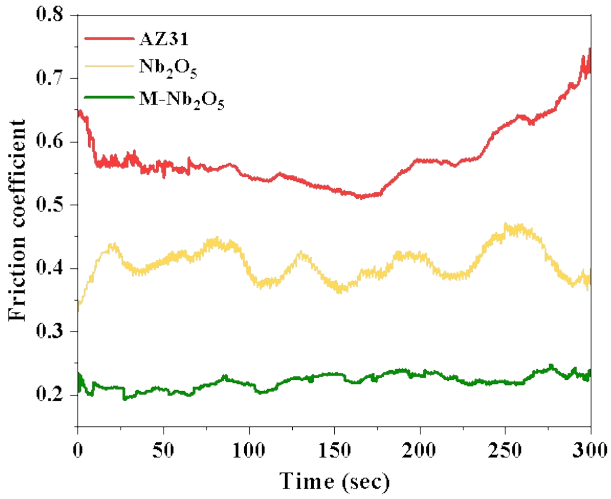

**Figure S8.** COF curves of untreated, $Nb_2O_5$, and M-$Nb_2O_5$ coated AZ31 samples.

**Table S1.** Deposition parameters of M-$Nb_2O_5$ multilayer coating.

| Deposition parameters | | Bottom layer (Mg) | Composite interlayer ($Nb_2O_5$-Mg) | Top layer ($Nb_2O_5$) |
|---|---|---|---|---|
| Sputtering Power (W) | Mg target | 60 | 15 | / |
| | $Nb_2O_5$ target | / | 200 | 250 |
| Deposition time (min) | | 10 | 320 | 210 |
| Ar Gas flow (sccm) | | 40 | | |
| Background pressure (Pa) | | $1\times10^{-3}$ | | |

**Table S2.** Performance parameters of layer and matrix materials.

| Materials properties | Young's modulus (GPa) | Poisson's ratio | Coefficient of thermal expansion ($10^{-6}$ /K) |
|---|---|---|---|
| AZ31[1] | 45 | 0.35 | 26.8 |
| Mg[2] | 44.8 | 0.35 | 25.2 |
| $Nb_2O_5$-Mg[3] | 52.4 | 0.275 | 15.5 |
| $Nb_2O_5$[4] | 60 | 0.2 | 5.8 |

## Reference

1 H. Hu, D. Zhang, F. Pan and M. Yang, *J Wuhan Univ Technol*, 2009, **24**, 376-382.
2 Taj, B. Prasanth Kumar and K. Vijayasree, *Mater. Today: Proc*, 2017, **4**, 9095-9099.
3 H. Guo, S. Gong and H. Xu, *Mater. Sci. Eng.A*, 2002, **325**, 261–269.
4 H. C. Chen, C. Y. Huang and P. W. Cheng, *Surf. Coat. Technol*, 2018, **344**, 449-457.

**Table 3S.** Corrosion parameters of naked and coated AZ31 specimens in SBF solution.

| Sample | $E_{corr}$ (V vs.Ag/AgCl) | $i_{corr}$ (A $cm^{-2}$) |
|---|---|---|
| AZ31 | -1.63 | $6.46\times10^{-5}$ |
| $Nb_2O_5$ | -1.54 | $3.43\times10^{-5}$ |
| M-$Nb_2O_5$ | -1.44 | $4.04\times10^{-6}$ |
